# Role of Myeloid-Derived Suppressor Cells in Amelioration of Experimental Autoimmune Hepatitis Following Activation of TRPV1 Receptors by Cannabidiol

**DOI:** 10.1371/journal.pone.0018281

**Published:** 2011-04-01

**Authors:** Venkatesh L. Hegde, Prakash S. Nagarkatti, Mitzi Nagarkatti

**Affiliations:** Department of Pathology, Microbiology and Immunology, University of South Carolina School of Medicine, Columbia, South Carolina, United States of America; Centre de Recherche Public de la Santé (CRP-Santé), Luxembourg

## Abstract

**Background:**

Myeloid-derived suppressor cells (MDSCs) are getting increased attention as one of the main regulatory cells of the immune system. They are induced at sites of inflammation and can potently suppress T cell functions. In the current study, we demonstrate how activation of TRPV1 vanilloid receptors can trigger MDSCs, which in turn, can inhibit inflammation and hepatitis.

**Methodology/Principal Findings:**

Polyclonal activation of T cells, following injection of concanavalin A (ConA), in C57BL/6 mice caused acute hepatitis, characterized by significant increase in aspartate transaminase (AST), induction of inflammatory cytokines, and infiltration of mononuclear cells in the liver, leading to severe liver injury. Administration of cannabidiol (CBD), a natural non-psychoactive cannabinoid, after ConA challenge, inhibited hepatitis in a dose-dependent manner, along with all of the associated inflammation markers. Phenotypic analysis of liver infiltrating cells showed that CBD-mediated suppression of hepatitis was associated with increased induction of arginase-expressing CD11b^+^Gr-1^+^ MDSCs. Purified CBD-induced MDSCs could effectively suppress T cell proliferation in vitro in arginase-dependent manner. Furthermore, adoptive transfer of purified MDSCs into naïve mice conferred significant protection from ConA-induced hepatitis. CBD failed to induce MDSCs and suppress hepatitis in the livers of vanilloid receptor-deficient mice (TRPV1^−/−^) thereby suggesting that CBD primarily acted via this receptor to induce MDSCs and suppress hepatitis. While MDSCs induced by CBD in liver consisted of granulocytic and monocytic subsets at a ratio of ∼2∶1, the monocytic MDSCs were more immunosuppressive compared to granulocytic MDSCs. The ability of CBD to induce MDSCs and suppress hepatitis was also demonstrable in Staphylococcal enterotoxin B-induced liver injury.

**Conclusions/Significance:**

This study demonstrates for the first time that MDSCs play a critical role in attenuating acute inflammation in the liver, and that agents such as CBD, which trigger MDSCs through activation of TRPV1 vanilloid receptors may constitute a novel therapeutic modality to treat inflammatory diseases.

## Introduction

Cannabidiol (CBD) is a major non-psychoactive cannabinoid component of marijuana (*Cannabis sativa*) [1]. CBD has been shown to have potent immunosuppressive and anti-inflammatory properties [2], [3], [4] and is currently approved for clinical use in some countries for the treatment of pain in multiple sclerosis (MS) patients [5]. In addition to MS, CBD has shown promise in several rodent models of inflammation [6]–[10]. A single dose of CBD has been shown to suppress serum TNF-α production induced by lipopolysaccharide (LPS) in mice and has been found to be beneficial in murine collagen-induced arthritis by inhibiting IFN-γ production and T cell proliferation [7].

Hepatitis is the inflammation of the liver that can be caused by various agents such as viruses, chemicals, drugs, alcohol, genetic factors, or the patient's own immune system [11]. The inflammation can be acute, flaring up and then resolving within a few weeks to months, or chronic, enduring over many years. Chronic hepatitis may simmer for 20 years or more before causing significant symptoms related to advanced liver damage such as cirrhosis (scarring and liver failure), liver cancer, or death. American liver foundation estimates that one in every 10 people in North America is afflicted with a liver, biliary or gallbladder disease.

Hepatitis represents a worldwide health problem in humans for which pharmacological treatments currently available are not adequate. Development of new drugs, however, requires proper animal models relevant to human hepatitis [12]. Majority of the liver diseases such as viral hepatitis, autoimmune hepatitis (AIH), primary biliary cirrhosis, primary sclerosing cholangitis, and liver allograft rejection are caused by activated T lymphocytes that infiltrate and destroy liver parenchyma leading to liver injury [13]. Injection of mice with the T-cell mitogenic plant lectin Concanavalin A (ConA), results in polyclonal activation of T lymphocytes leading to liver selective inflammatory response, which mimics activated T-cell mediated hepatitis [14]. ConA-induced hepatitis has been well established as an ideal animal model to study T-cell mediated hepatic injury and has been used extensively to elucidate various aspects of human T cell-mediated liver diseases, such as AIH and viral hepatitis [14]–[20]. It is characterized by elevated levels of aspartate transaminase (AST) and alanine transaminase (ALT) enzyme activities, and inflammatory cytokines in blood and liver. Histologically, ConA injection induces dramatic inflammatory infiltrates in the liver, particularly T cells. In this model, liver injury occurs without sensitization or priming as compared to other models of liver inflammation such as galactosamine-lipopolysaccharide (LPS) induced hepatitis [21].

Myeloid-derived suppressor cells (MDSCs), are a newly identified suppressor cells of myeloid lineage which co-express CD11b and Gr-1 antigens [22], [23]. These cells, originally identified in tumor bearing hosts, have been shown to possess potent suppressive functions and regulate inflammatory responses [24], [25]. Although, cannabidiol is known to be highly immunosuppressive and anti-inflammatory, its effect on this important suppressive cell population has not been investigated.

Autoimmune hepatitis is generally treated with medications that suppress the immune system, such as prednisone and azathioprine, although these treatments are not universally effective and long term side effects exist [26], [27]. New treatments, vaccines, and prevention strategies for hepatitis continue to emerge. Here, we describe our finding that a single dose of CBD is effective in significantly suppressing ConA-induced T cell-mediated hepatic inflammation in mice. Importantly, we have identified a novel pathway through which CBD suppresses hepatitis involving the induction of MDSCs in liver following activation of vanilloid receptor, TRPV1. These observations will help in developing CBD as a potential drug to treat inflammatory liver diseases.

## Results

### Cannabidiol suppresses ConA-induced hepatitis

**Figure 1:**
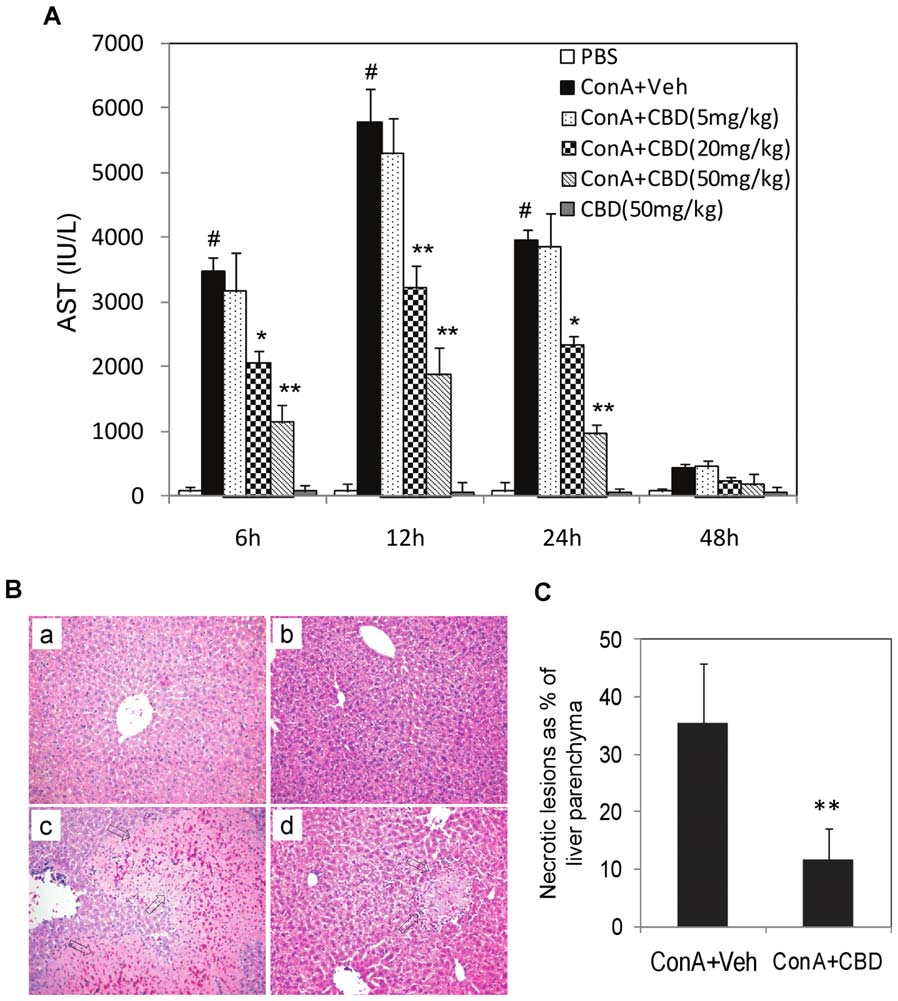
Cannabidiol (CBD) attenuates ConA-induced hepatitis in mice. WT mice (n = 6) were injected with PBS (control), or ConA (12.5 mg/kg bd.wt., *i.v.*) to induce hepatitis. ConA injected mice were administered *i.p.* with vehicle or CBD at 5, 20 or 50 mg/kg bd.wt. CBD group received CBD alone at 50 mg/kg. A) Blood was collected at 6, 12, 24 and 48 h and serum aspartate transaminase (AST) was determined by spectrophotometric assay. Data represent mean ± SEM from 6 individual mice. The changes in AST levels were analyzed by Student's *t*-test (# p<0.01 compared to PBS; *p<0.05, **p<0.01 compared to ConA+veh). **Histological analysis:** B. Representative H&E stained liver sections obtained 24 h post ConA injection in WT mice (original magnification ×100). a) Vehicle and b) CBD alone, show normal tissue morphology; c) ConA+veh shows large necrotic lesion induced by ConA (arrows); d) ConA+CBD (50 mg/kg bd.wt.) shows less necrosis (arrows) of liver parenchyma. C) Quantification of necrotic lesions as a percentage of liver parenchyma. Data are mean ± SEM from 4 mice per treatment. At least five fields were analyzed per section from each liver sample. PBS and CBD controls did not show any necrotic lesions. Student's *t*-test, **p<0.01 compared to ConA.

A single injection of concanavalin-A (ConA) has been shown to induce hepatitis in mice mimicking the symptoms of human autoimmune hepatitis [14], [16]–[18]. Acute liver inflammation occurs within 8–24 h of injecting ConA, with clinical and histological evidence of hepatitis, elevation of transaminase activities in the plasma and hepatic inflammatory lesions characterized by massive leukocyte accumulation and hepatic necrosis. In this study, we investigated if CBD can be used to treat hepatitis using this model. WT mice were injected with PBS (vehicle), or ConA to induce hepatitis. ConA injected mice were administered (*i.p.*) with vehicle (ConA+veh group) or different doses of CBD, ranging from 5 mg/kg to 50 mg/kg body wt (ConA+CBD groups), 5 minutes after ConA injection. Some mice received CBD alone at the maximum dose of 50 mg/kg (CBD group). Next, blood was collected at 6, 12, 24 and 48 h and plasma AST (aspartate transaminase) was determined by spectrophotometry using AST assay kit, as described [28]. As shown in Fig. 1A, intravenous injection of ConA resulted in dramatic increase in plasma AST levels over vehicle control, indicative of acute hepatitis. Increased AST levels were seen as early as 6 h after ConA injection, reaching a peak around 12 h and declining thereafter. At 48 h, the plasma AST reached normal levels. Mice which received both ConA and cannabidiol (ConA+CBD) showed significantly less plasma AST activity compared to ConA-injected (ConA+veh) group demonstrating reduced liver injury upon CBD treatment. Cannabidiol alone injected at the maximum dose showed AST levels similar to that of vehicle control at all-time points tested thereby suggesting that CBD did not mediate any direct hepatotoxic effects.

Histological examination of paraformaldehyde fixed liver sections was performed. Vehicle and CBD alone injected groups showed normal tissue morphology and did not show any signs of liver inflammation. Significant leukocyte infiltration and tissue necrosis was observed 24 h after ConA-injection (Fig. 1B). Although, CBD treated groups (ConA+CBD) still had significant cellular infiltrates, CBD treatment resulted in marked decrease in liver tissue injury with a significant decrease in necrotic lesions (Fig.1B, C), thereby corroborating that CBD was very effective in protecting against ConA-induced autoimmune liver injury.

### Cannabidiol suppresses pro-inflammatory cytokines

**Figure 2:**
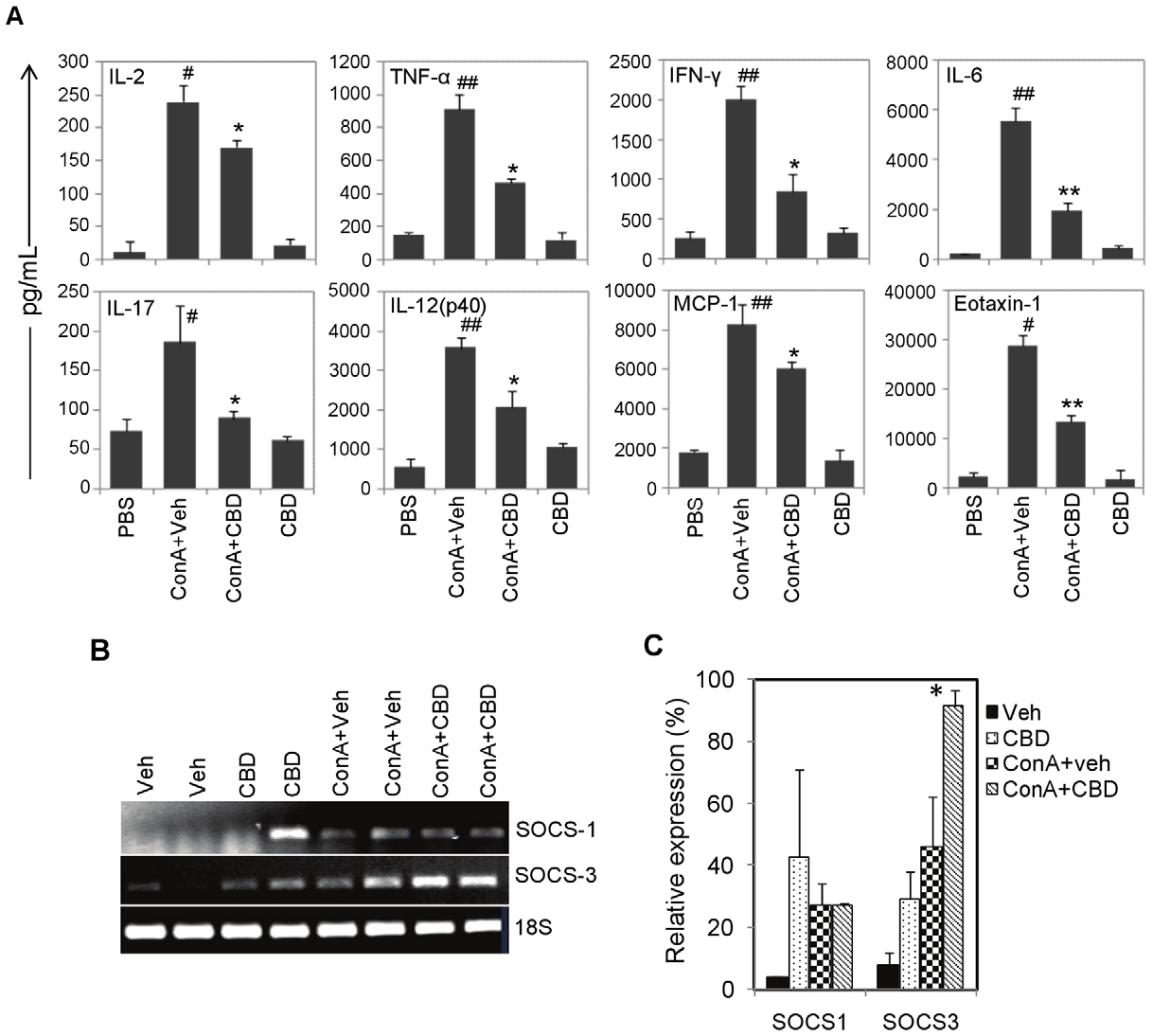
Analysis of inflammatory cytokines, SOCS1 and SOCS3. A) CBD acts by suppressing pro-inflammatory cytokines in ConA-induced hepatitis. WT mice (n = 6) were injected with PBS or ConA. ConA injected mice were administered with vehicle or CBD (25 mg/kg bd.wt.). CBD group received CBD alone at 25 mg/kg bd.wt. Blood was collected after 12 h and serum cytokines were determined by Bioplex assay. Error bars represent mean ± SEM (n = 6). The data were analyzed by Student's *t*-test (^##^p<0.01 compared to PBS; *p<0.05, **p<0.01 compared to ConA+Veh). B) Semi-quantitative RT-PCR for SOCS-1 and SOCS-3 in livers 2 h after various treatments *in vivo*. 18S was used as a loading control. C. The densities of bands were quantified using gel imaging system (BioRad) and expressed as percentage expression relative to 18S. Student's *t*-test, *p<0.05 compared to ConA.

Several cytokines and chemokines were analyzed in the serum of mice 12 h after ConA-challenge using multiplex cytokine array system. This time point was selected because AST levels peak at around 12 h (Fig. 1A). ConA+veh injection resulted in significant increase in the levels of proinflammatory cytokines, predominantly IL-2, TNF-α, IFN-γ, IL-6, IL-12(p-40), IL-17, MCP-1 and eotaxin-1 (CCL11) (Fig. 2A) compared to vehicle control. The levels of these pro-inflammatory cytokines were significantly decreased in ConA+CBD mice, demonstrating that CBD treatment led to effective suppression of multiple inflammatory cytokines which may afford protection against hepatocellular damage.

We also determined the levels of mRNA for suppressor of cytokine signaling 1 and 3 (SOCS-1 and SOCS-3) at an early time point (2 h) in livers by semi-quantitative RTPCR. Although, there was no significant difference in SOCS-1 mRNA levels, SOCS-3 was significantly induced in ConA+CBD injected mice when compared to ConA+veh injected mice (Fig. 2B &C), suggesting a role for SOCS-3 mediated mechanism in the suppression of cytokines by CBD during hepatitis.

### Analysis of liver infiltrating cells

**Figure 3:**
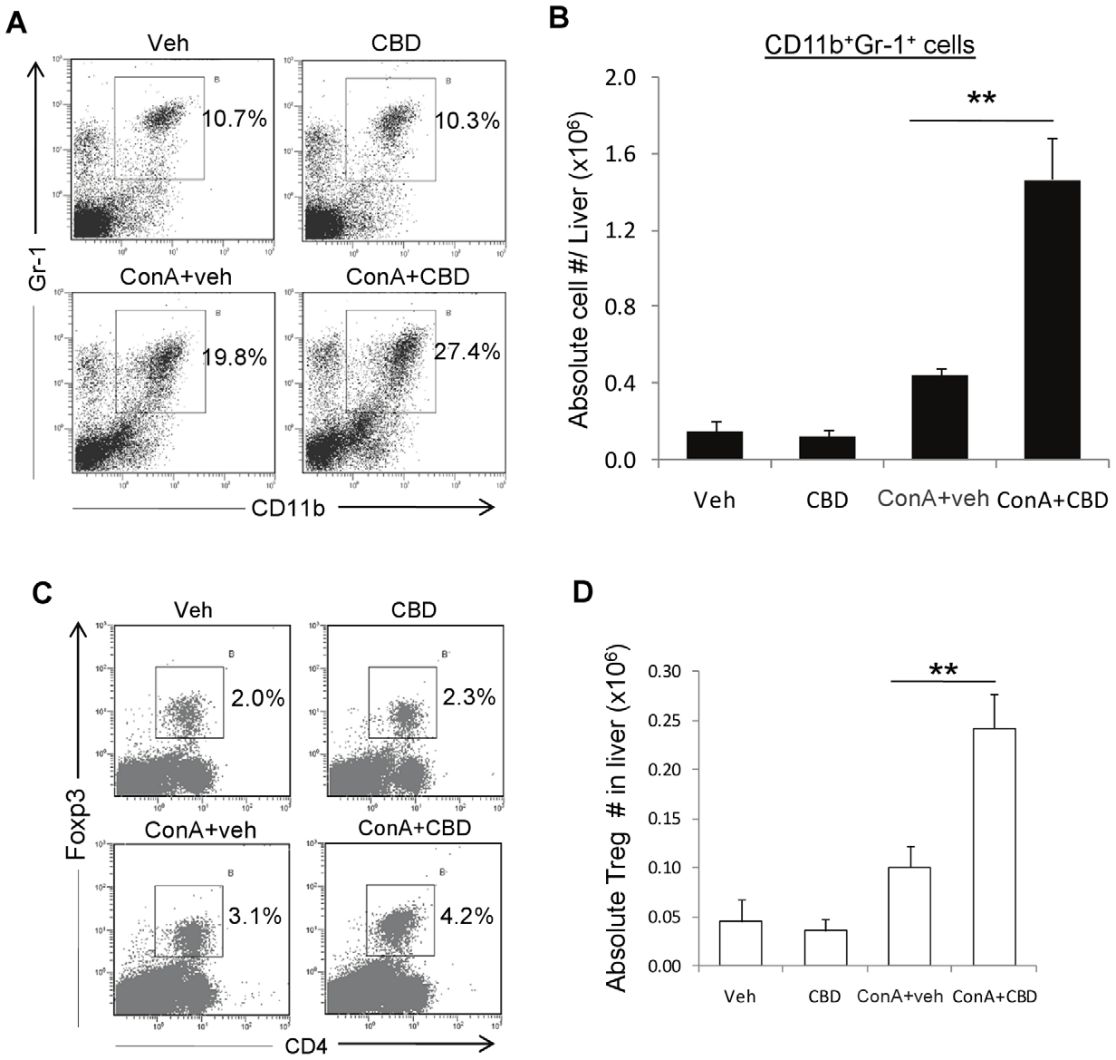
Phenotypic characterization of liver infiltrating cells: CBD treatment of hepatitis increases predominantly CD11b^+^Gr-1^+^ cell numbers in liver. WT mice were injected with PBS (Veh) or ConA. ConA injected mice were simultaneously administered (*i.p.*) with vehicle or CBD (25 mg/kg bd.wt.). CBD group received CBD alone at 25 mg/kg bd.wt. Liver infiltrating cells isolated after 12 h were stained for CD11b and Gr-1, and analyzed by FACS. Representative flow profiles for each sample showing frequency of CD11b^+^Gr-1^+^ cells (gated) is shown in panel A. Absolute numbers of CD11b^+^Gr-1^+^ cells were calculated from percentages and total cell numbers per liver (B). Cells were also stained and analyzed for Foxp3^+^ Tregs (D, E). Data represent mean ± SEM from 4 mice per treatment. Data were analyzed by Student's *t*-test (*p<0.05, **p<0.01).

To understand the cellular mechanisms involved, we isolated the liver infiltrating cells and subjected them to phenotypic characterization. While performing these studies, we noted that the number of T cells or mature macrophages (F4/80^high^) did not show any significant change following CBD treatment (data not shown). However, we noticed a dramatic increase in the percentage and absolute numbers of cells expressing CD11b and Gr-1. In-depth analysis revealed that vehicle-treated mice had significant (∼10%) CD11b^+^Gr-1^+^ in the liver and CBD treatment alone did not affect this percentage or the absolute number (Fig. 3A, B). Administration of ConA caused an increase in both the percentage and absolute number of CD11b^+^Gr-1^+^ cells. ConA+CBD treatment caused a further robust induction of CD11b^+^Gr-1^+^ cells when compared to ConA+veh treatment group. We also noted that ConA+CBD treatment caused a relatively modest increase in CD4^+^Foxp3^+^ Tregs (Fig. 3C, D) in liver when compared to ConA+veh group indicating that CBD acts by predominantly inducing CD11b^+^Gr-1^+^ cells in liver.

### Identifying CD11b^+^Gr-1^+^ in the liver as MDSCs

Recently, myeloid-derived suppressor cells (MDSCs) that express CD11b and Gr-1 antigens have been shown to be induced at sites of inflammation that help down-regulate immune responses [23], [25], [29], [30]–[32]. The increased presence of CD11b^+^Gr-1^+^ cells in mice with hepatitis suggested that such cells may represent immunosuppressive MDSCs and that they may play a critical role in suppressing the acute inflammation and liver injury as evidenced by the AST levels reaching normal levels by 48 h (Fig. 1A). Furthermore, CBD may promote the induction of such cells thereby further protecting the host from acute hepatitis.

**Figure 4:**
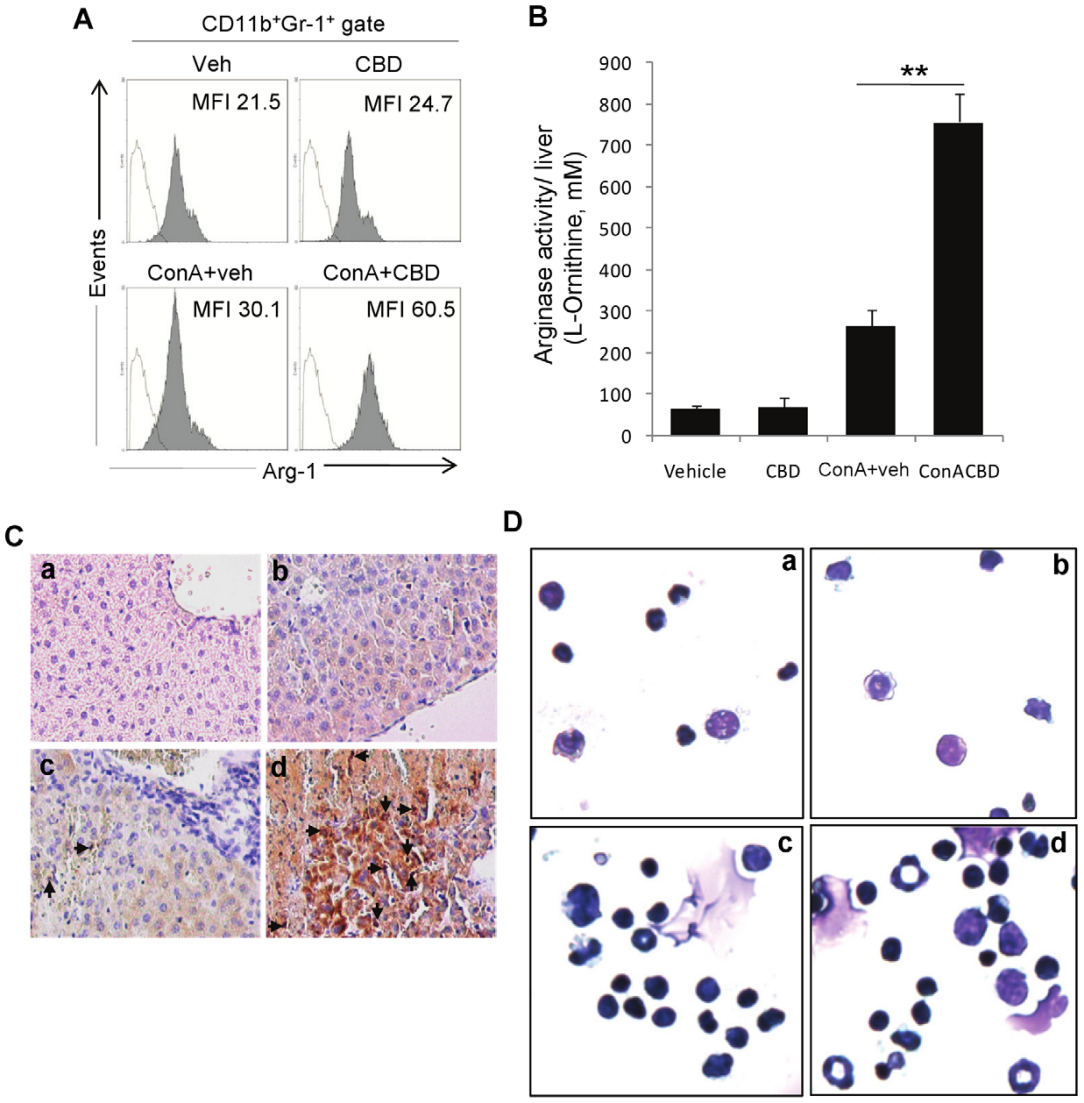
Characterization of MDSCs in liver. Liver infiltrating cells isolated from indicated treatment groups (n = 4) were triple-stained for CD11b, Gr-1 and intracellular arginase 1. Respective histograms (filled) for arginase expression on CD11b^+^Gr-1^+^ gated cells are shown for each group with respective MFI (A). Open histograms represent staining control. B) Arginase functional activity was determined by spectrophotometric assay using lysates of liver infiltrating cells from each group. Data are mean ± SEM (n = 4). Student's *t*-test, **p<0.01. C) Immunohistochemistry for arginase expression in liver sections and D) Morphology of infiltrating liver cells by Wright Giemsa staining (a. Veh, b. CBD, c. ConA+veh, d. ConA+CBD).

In order to characterize the CD11b^+^Gr-1^+^ cells found in the liver as MDSCs, we triple-stained the isolated infiltrating cells for CD11b, Gr-1, and for the expression of intracellular arginase 1 (Arg1), which is one of the characteristic features of MDSCs (Fig. 4A). We found that CD11b^+^Gr-1^+^ cells in all groups were Arg-1^+^. However, CD11b^+^Gr-1^+^ cells from ConA+CBD group showed increased Arg-1 expression as indicated by higher mean fluorescence intensity (MFI). There was also a significant increase in arginase functional activity in infiltrating cells isolated from livers of ConA+CBD group as compared to ConA+veh group (Fig. 4B). Immunohistochemistry for Arg-1 expression in liver sections revealed large number of positively stained cells in ConA+CBD injected mice (Fig. 4C). Wright-Giemsa staining of cytospin preparations of Percoll isolated infiltrating liver cells (Fig. 4D) showed granulocytic type cells with circular nuclei as well as monocytic type cells in both ConA+veh and ConA+CBD groups, although the frequency of such cells was higher in ConA+CBD group.

### CBD-induced liver CD11b^+^Gr-1^+^ MDSCs are immunosuppressive *in vitro* and *in vivo*

**Figure 5:**
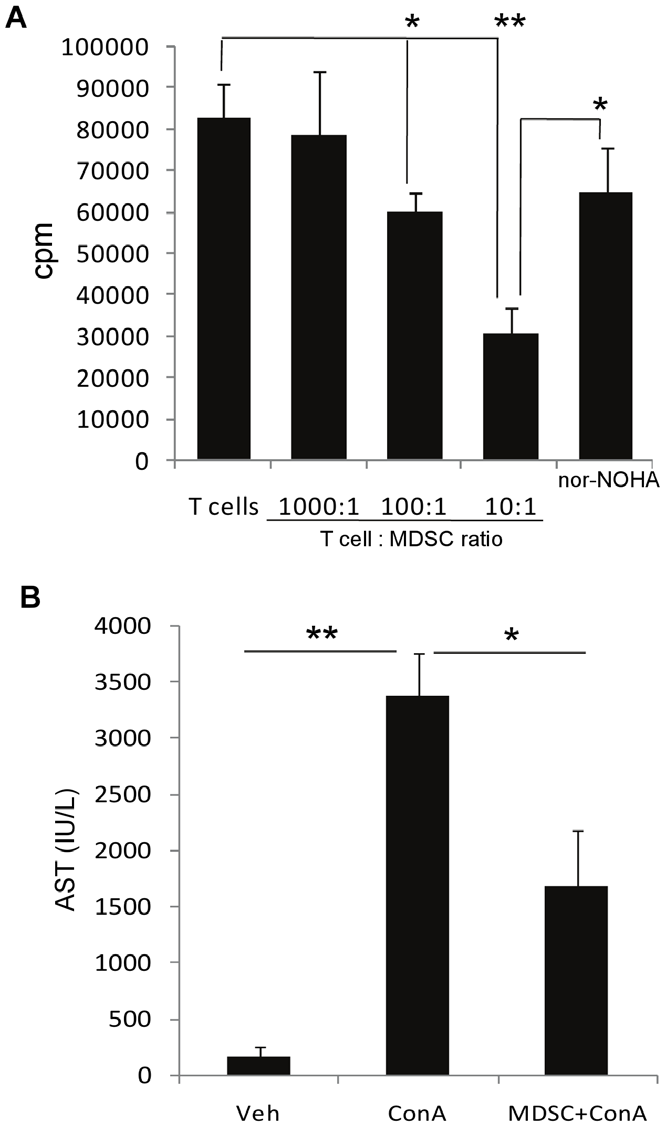
CBD-induced MDSCs in liver are immunosuppressive *in vitro* and *in vivo.* CD11b^+^Gr-1^+^ cells from liver infiltrating cells of ConA+CBD injected mice were purified, irradiated and co-cultured at different ratios with naïve syngenic purified T cells in the presence of mitogen ConA (4 µg/mL) for 48 h. T cell proliferation was determined by [3H]thymidine incorporation during the last 8 h of culture. In some wells with T cell: MDSC ratio of 10∶1, arginase-1 inhibitor (nor-NOHA) was added at the start of the culture at a concentration of 100 µM. Data are mean ± SEM of triplicate determinations and representative of two independent experiments. (B) WT mice (n = 4) were injected with vehicle or ConA. Five million purified CD11b^+^Gr-1^+^ cells isolated from livers of ConA+CBD group, were adoptively transferred 12 h before injecting ConA. Blood was collected 12 h post-ConA injection and analyzed for AST. Data represent mean ± SEM from 4 mice per treatment. Student's *t-*test (*p<0.05, **p<0.01).

To assess the immunosuppressive activity of CD11b^+^Gr-1^+^ MDSCs induced by CBD in livers, we analyzed them for their ability to suppress T cell proliferation. To this end, we sorted CD11b^+^Gr-1^+^ cells (>90% purity) from the livers of mice injected with ConA+CBD, irradiated them (2000 rad) and co-cultured at different ratios with purified syngeneic lymph node T cells in the presence of mitogen ConA (4 µg/mL) for 48 h. T cell proliferation was determined by thymidine incorporation during the last 8 h of culture. CBD-induced CD11b^+^Gr-1^+^ cells from liver significantly suppressed T cell proliferation at 100∶1 and 10∶1 ratios of T cell: MDSC (Fig. 5A). In some wells with T cell: MDSC ratio of 10∶1, arginase-1 inhibitor (nor-NOHA) was added at the start of the culture. As can be seen, co-incubation with arginase inhibitor significantly reversed the suppression of T cell proliferation induced by MDSCs. Together, these data conclusively demonstrated that the CD11b^+^Gr-1^+^ cells found in the livers were actually immunsuppressive MDSCs.

To further test if CBD-induced MDSCs can suppress liver injury, we adoptively transferred purified MDSCs into naïve mice before challenging them with ConA. Five million CD11b^+^Gr-1^+^ cells isolated from livers of ConA+CBD group were injected into naïve mice, followed by ConA 12 h later. The transferred MDSCs were able to significantly protect against liver injury as indicated by decreased AST levels (Fig. 5B). These data showed that the CBD-induced MDSCs exhibit immunosuppressive functions *in vivo* and that they can prevent acute liver injury.

### Role of vanilloid receptors (TRPV1) in CBD-mediated suppression of liver injury

**Figure 6:**
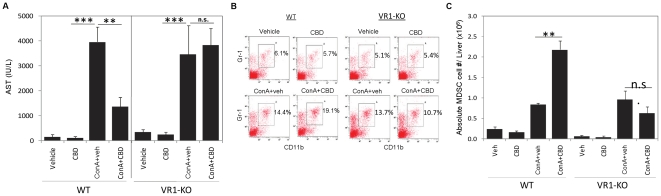
Role of TRPV1 (vanilloid) receptors. WT or TRPV1 vanilloid receptor knockout (VR1-KO) mice were injected with vehicle, CBD, ConA+veh or ConA+CBD. A) After 16 h, blood was collected and serum AST levels were measured. B) Liver infiltrating leukocytes were stained and analyzed for CD11b^+^Gr-1^+^ MDSCs by FACS. Representative dot plots with percentage of MDSCs shown (gated). C) Absolute number of MDSCs in the liver of WT and VR1-KO mice. Data are mean ± SEM with 4 mice per treatment. **p<0.01, ***p<0.001, n.s. (not significant) based on Student's *t*-test.

**Figure 7:**
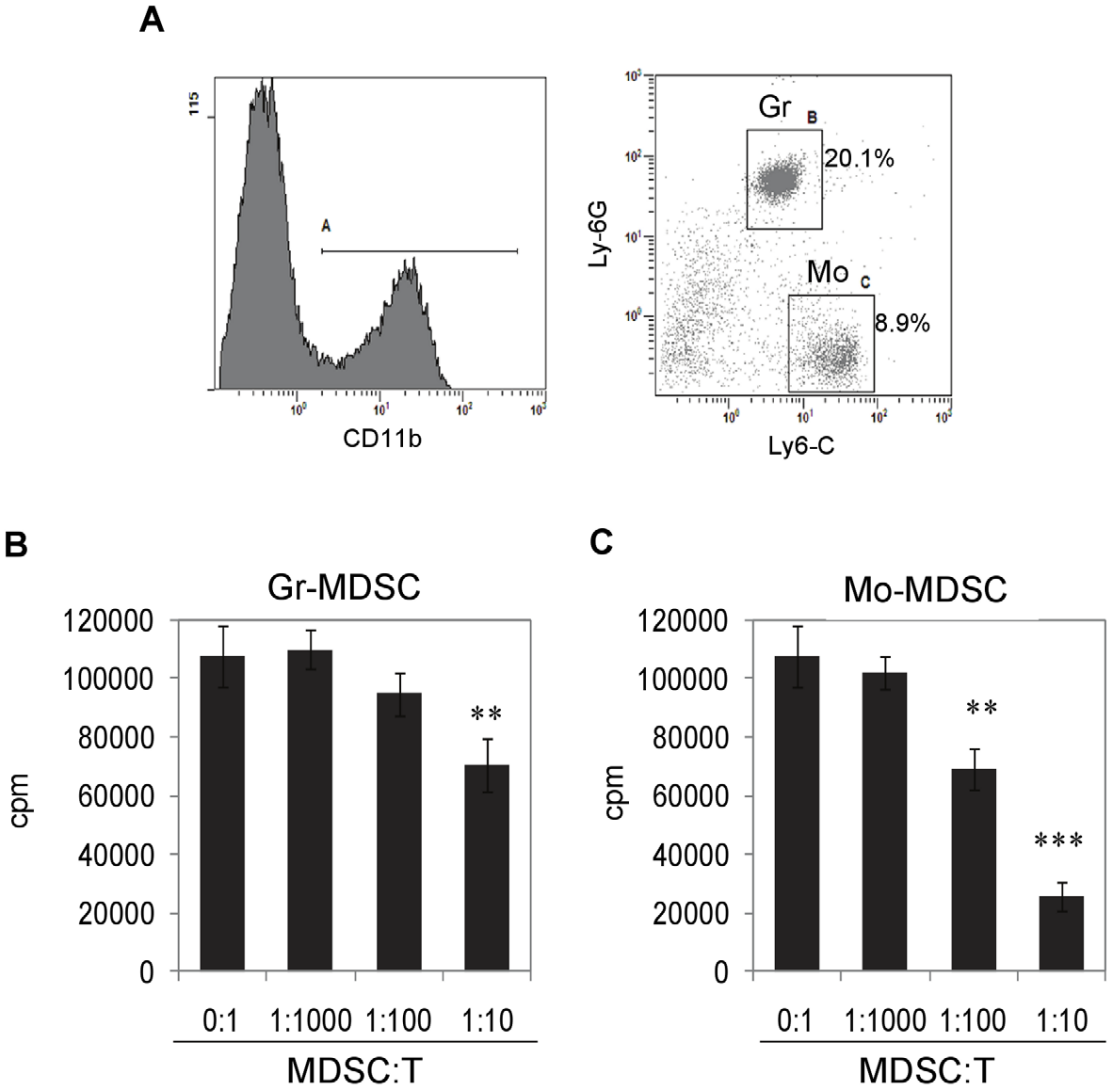
Identification of MDSC subsets induced by CBD in liver and their suppressive activity. Liver infiltrating cells were isolated from ConA+CBD injected mice at 12 h. Cells were triple-stained for CD11b, Ly6-G and Ly-6C, and analyzed by FACS. A) Cells were gated for CD11b^+^ expression (histogram) and further analyzed for Ly6-G and Ly6-C antigens (dot plot). Representative dot plot shows percentages of CD11b^+^Ly6-G^+^Ly6-C^+^ granulocytic MDSCs (Gr-MDSC) and CD11b^+^Ly6-G^–^Ly6-C^+^ monocytic MDSCs (Mo-MDSC). B & C) MDSC subsets were purified by FACS sorting and used in T cell proliferation assay *in vitro* at different ratios with syngenic purified T cells stimulated with ConA. T cell proliferation was assessed by [3H]thymidine incorporation at 48 h. T cells cultured without MDSCs and with ConA served as the positive control. Data are mean ± SEM for triplicate determinations and representative of two separate experiments. **p<0.01, ***p<0.001, based on Student's t test.

Next, we addressed the mechanism of action of CBD. CBD has been shown to primarily function through vanilloid receptors (transient receptor potential vanilloid1, TRPV1) [33], [34]. To test the role of TRPV1 in this model, we used vanilloid receptor knockout (TRPV1^−/−^ or VR1-KO) mice. VR1-KO mice developed hepatitis in response to ConA as indicated by increase in AST levels, which was similar to that seen in ConA-injected wild-type (WT) mice (Fig. 6A). However, unlike in WT mice, CBD was not able to suppress AST levels in VR1-KO mice, suggesting a critical role for TRPV1 in mediating the anti-inflammatory activity of CBD. Moreover, when we enumerated the number of MDSCs in the liver in this experiment, CBD was able to induce MDSCs in ConA-injected WT but not VR1-KO mice (Fig. 7B & C), thereby suggesting that induction of MDSCs by CBD in the livers of hepatitis mice was dependent on TRPV1 receptors. It was interesting to note that administration of ConA alone also induced lower levels of MDSCs in the liver which was similar in both WT and VR1-KO mice, suggesting that this response was independent of TRPV1 receptor.

### Analysis of MDSC subsets

The CD11b^+^Gr-1^+^ MDSCs are known to contain heterogeneous mixture of myeloid cells with suppressive function. Recently, two major subsets of MDSCs have been identified based on the expression of CD11b, Ly6-G and Ly6-C antigens. Granulocytic subsets (Gr-MDSC) express both Ly6-G and Ly6-C along with CD11b (CD11b^+^Ly6-G^+^Ly6-C^+(int)^) while monocytic subsets (Mo-MDSC) express only Ly6-C and CD11b (CD11b^+^Ly6-G^–^Ly6-C^+^) [29], [35], [36]. To identify these subsets, we used mAbs specific to Ly6-G (clone: 1A8) and Ly6-C (clone: HK1.4). Infiltrating cells from the liver of CBD-treated hepatitis mice showed significant frequency of CD11b^+^Ly6-G^+^Ly6C^+(int)^ granulocytic and CD11b^+^Ly6-G^–^Ly6C^+^ monocytic MDSCs in close to 2∶1 ratio (Fig. 7A). These subsets were purified by sorting and used in T cell proliferation assay to determine their relative suppressive potential. While both the subsets significantly suppressed T cell proliferation *in vitro* (Fig. 7B & C), Mo-MDSCs were highly immunosuppressive compared to Gr-MDSCs.

### CBD attenuates SEB-induced acute liver injury

**Figure 8:**
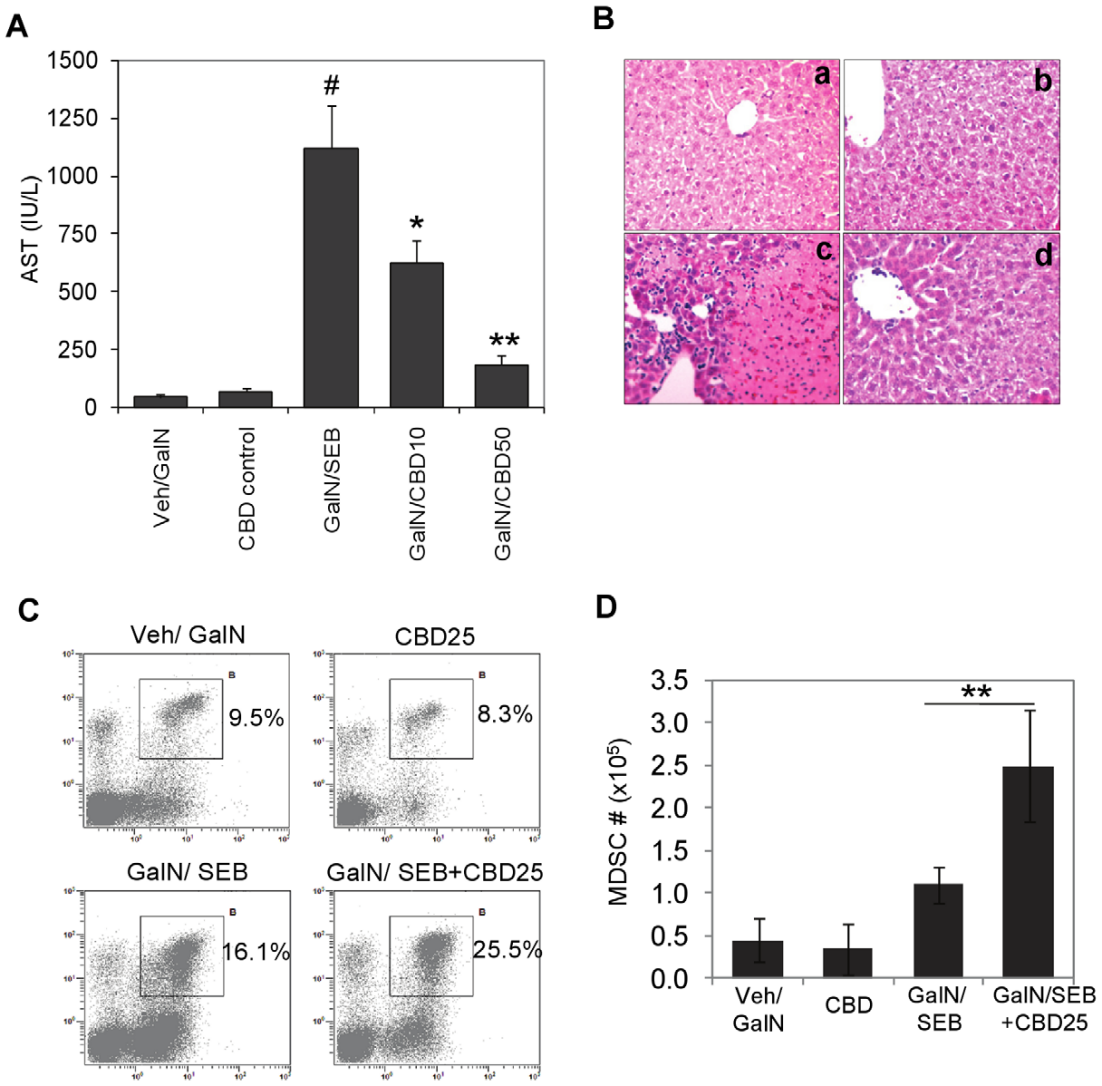
CBD attenuates SEB-induced acute liver injury by inducing MDSCs in liver. WT mice (n = 6) were sensitized by injecting D-galactosamine (GalN), 20 mg/mouse in 100 µL PBS (*i.p.*). After 15 min, SEB was injected (40 µg/mouse, in 100 µL PBS, *i.p.*). For treatment groups, 10 or 50 mg/kg bd.wt. of CBD was administered *i.p.* A) Blood was collected at 8 h post SEB injection and serum AST levels were measured. Student's *t*-test (^#^p<0.01 compared to Veh/GalN control; *p<0.05, **p<0.01 compared to GalN/SEB). B. **Histology:** Liver samples after 48 h were fixed and embedded in paraffin. Five µm sections were stained by H&E and analyzed by light microscopy. Representative photomicrographs are shown. a) Veh/GalN, b) CBD50, c) GalN/SEB and d) GalN/SEB+CBD50 (CBD50 = 50 mg/kg). Liver infiltrating cells from the treatment groups as indicated were isolated at 24 h and stained for CD11b and Gr-1 and analyzed by flow cytometry (CBD25 = 25 mg/kg). Representative dot plots are shown with percentages of CD11b^+^Gr-1^+^ MDSCs (C). D) Absolute number of MDSCs in liver for each group (n = 4). Student's *t*-test (**p<0.01 compared to GalN/SEB).

We sought to see if the suppressive effect of CBD was specific to ConA-induced liver inflammation or would it work in any other acute liver inflammation model. To this end, we used Staphylococcal Enterotoxin B (SEB)-induced acute liver inflammation. Injection of SEB into GalN-sensitized mice led to increased AST levels at 12 h, indicative of acute hepatitis (Fig. 8A). CBD was able to decrease the AST levels significantly in a dose dependent manner, showing that CBD was effective in suppressing liver inflammation in this model. Moreover, in this model as well, CBD treatment of hepatitis was associated with significant increase in the frequency and number of CD11b^+^Gr-1^+^ MDSCs in liver (Fig. 8B & C).

## Discussion

ConA-induced hepatitis is a well-established model for hepatitis caused as a consequence of T and NKT cell activation [14], [37]. In the current study we demonstrate for the first time that CBD, a non-psychoactive cannabinoid, can significantly reduce ConA-induced inflammation and protect the mice from acute liver injury, as indicated by marked decrease in plasma AST levels and necrotic lesions. We observed that a single dose of CBD as low as 20 mg/kg body weight was effective in this model. CBD is already approved for clinical use in Canada, in combination with THC under the trade name Sativex (GW Pharmaceuticals) to alleviate neuropathic pain, spasticity and overactive bladder in multiple sclerosis and also prescribed for cancer patients to reduce nausea and improve appetite [5], [38]. CBD is also in clinical trials to reduce schizophrenic symptoms [39], [40]. The daily recommended dose of Sativex is 5 oral sprays per day which is equivalent to 12.5 mg CBD per day as a long term treatment. In one of the earliest double-blind studies on CBD, normal volunteers received 3 mg/kg daily CBD for 30 days and epileptic patients received 200–300 mg per day for up to 4 1/2 months without any signs of toxicity or serious side effects [41]. The study concluded that CBD was effective as an anti-epileptic drug or as a potentiating agent for other epileptic drugs compared to placebo. In another randomized double-blind controlled study of Huntington disease patients, CBD was given orally at an average daily dose of 700 mg/day for six weeks, where it was found neither symptomatically effective nor toxic relative to placebo [42]. In the current study, we used a single dose of CBD at 20–50 mg/kg body weight in mice, which showed significant efficacy in an acute inflammation model. This dose converts to 1.6–4.1 mg/kg of human equivalent dose (HUD) based on body surface area normalization (BSA) method [43], and translates to a single dose of 96–246 mg in an average individual of 60 kg, which seems to be safe and acceptable dose based on several previous studies in humans mentioned above.

ConA-induced hepatitis is primarily mediated by activated T cells and NKT cells, and the induction of hepatitis is associated with the surge in the production of various pro-inflammatory cytokines. TNF-α and IFN-γ are the first cytokines produced after ConA injection, and are the most critical in inducing hepatitis as anti-TNF and anti-IFN-γ antibodies confer protection against the disease [16], [44]. We found that CBD treatment resulted in suppression of various pro-inflammatory cytokines including TNFα and IFN-γ induced by ConA.

The protective effect of SOCS3 in liver inflammation is known [45]. Replenishing the intracellular stores of SOCS3 with cell penetrating forms of SOCS3 has been shown to effectively suppress the devastating effects of acute inflammation in ConA, LPS or SEB-induced hepatitis models [45]. Attenuated liver injury in STAT1^–/–^ and IFN-γ^–/–^ mice in response to ConA was associated with enhanced SOCS3 activation Whereas, decreased SOCS3 activation in IL-6^–/–^ mice seem to result in enhanced hepatitis in response to ConA [46]. Furthermore, forced expression of SOCS3 in T cells has been shown to protect against the development of ConA-induced hepatitis [47]. In the current study, we observed that CBD induced SOCS-3 in the liver after ConA challenge which may contribute to suppression of inflammatory cytokines observed, and decreased liver injury.

CBD treatment caused a dramatic decrease in inflammatory loci or necrotic lesions in livers of ConA-treated mice. Our phenotypic analysis and detection of CD11b^+^Gr-1^+^ cells in livers showed that majority of the infiltrating cells in the CBD treated group consisted of MDSCs. MDSCs express arginase which can metabolize and deplete L-Arginine, an essential amino acid required for the proliferation and function of T cells. This seems to be the primary mechanism by which MDSCs suppress activated T cells [23], [30], [48]. In the current study, using arginase enzyme activity assay based on the conversion of L-arginine to L-ornithine, we demonstrated that CBD-induced MDSCs in liver expressed functionally active arginase. Purified MDSCs from CBD treated mice were able to suppress ConA-stimulated T cell proliferation *in vitro* in Arg-1 dependent manner. *In vivo,* the suppressive activity of adoptively transferred MDSCs could be demonstrated in models of inflammation [49] and cancer [50]. In the current model, we showed that the adoptively transferred MDSCs induced by CBD were able to significantly protect mice from ConA-hepatitis, thereby conclusively demonstrating that CBD-induced MDSCs were indeed functional and can suppress hepatitis. It should be noted that ConA-induced hepatitis by itself showed a small increase in the number of MDSCs in livers. This may be a natural mechanism following inflammatory stimuli to regulate inflammation. Such MDSCs may play a crucial role in helping the host recover from inflammation as evidenced by the restoration of AST levels to basal levels by 48 h. Nevertheless, CBD treatment further triggered the induction of MDSCs which also expressed higher density of Arg-1. It is interesting to note that CBD alone did not induce any MDSCs in liver of naïve mice. We speculate that CBD triggers the induction of MDSCs only when there is an insult or inflammation in the liver.

Cannabidiol has been shown to bind and function primarily through TRPV1 or vanilloid receptors [33], [34]. Vanilloid receptors mediate anti-hyperalgesic effect of CBD, in a rat model of acute inflammation [34]. TRPV1 ion channels also mediate the response to painful heat, extracellular acidosis, and capsaicin, the pungent compound from plants which upon prolonged use decreases TRPV1 activity by a phenomenon called desensitization [51]. CBD possesses no, or very weak affinity for the central and peripheral canabbinoid receptors (CB1 and CB2) and is not psychoactive [33], [52]. Use of vanilloid receptor knock-out mice in our study clearly showed that CBD induced suppression of inflammation in ConA-hepatitis was dependent on TRPV1, so was the induction of MDSCs by CBD in the livers of ConA-injected mice. Recently, we demonstrated that activation of cannabinoid receptors can trigger massive induction of immunosuppressive MDSCs [53]. We noted this was dependent on the production of G-CSF. In the current study, we tested if some specific chemokine or cytokine may be involved in TRPV1-mediated induction or accumulation of MDSCs by CBD in liver. Our attempt to identify such factor, particularly looking at GM-CSF, G-CSF, and KC by ELISA was not conclusive (data not shown). Even though there was a significant increase in G-CSF 24 h after CBD treatment of hepatitis corresponding with increase in number of MDSCs in liver as well as decrease in liver enzymes (inflammatory marker), blocking studies with anti-G-CSF Ab failed to reverse the CBD effect.

Finally, we showed that CBD-induced suppression of acute liver inflammation is not specific to ConA-induced hepatitis, but it is also equally effective in other acute hepatitis models such as sensitization with GalN followed by induction of liver inflammation by sub lethal doses of SEB. Overall, the current study demonstrates that MDSCs may play a critical role in protecting the liver from acute inflammation. The most interesting observation in this study was robust induction of CD11b^+^Gr-1^+^ MDSCs by CBD in the livers of ConA-induced mice that were immunosuppressive, which protected mice from hepatitis upon adoptive transfer. Moreover, CBD was found to induce MDSCs following activation of TRPV1 inasmuch as, CBD failed to trigger MDSCs in the livers of TRPV1 deficient mice and failed to protect them from hepatitis. Together, these studies not only demonstrate that CBD can protect the host from acute liver injury but also provide evidence for the first time that MDSCs may play a critical role in protecting the liver from acute inflammation. Non-psychoactive cannabinoids such as CBD possess great therapeutic potential in treating various inflammatory liver diseases, including autoimmune hepatitis.

## Materials and Methods

### Mice

Female C57BL/6 mice (8–12 weeks) were obtained from National Cancer Institute (Frederick, MD). Female vanilloid receptor knockout (TRPV1) mice on BL/6 background were purchased from the Jackson labs (Bar Harbor, ME, USA).

### Ethics Statement

Mice were housed and maintained under specific pathogen-free conditions in the Animal Resource Facility of University of South Carolina and all experiments were pre-approved by the Institutional Animal Care and Use Committee (ICAUC approval number AUP # 1620).

### Reagents

Cell culture grade concanavalin A was from Sigma-Aldrich (St. Louis, MO). The monoclonal antibodies (mAb), FITC-conjugated anti-CD11b and anti-Ly6-C (clone: HK1.4), PE-conjugated anti-CD3, anti-Gr-1 and anti-F4/80, AlexaFlour647-conjugated CD11b, and purified anti-CD16/CD32 were from eBioscience. PE-conjugated anti-Ly6-G (clone: IA-8) was from BD Bioscience. Cannabidiol was provided by NIDA, NIH. N^ω^-hydroxy-nor-Arginine (nor-NOHA) was obtained from Cayman Chemical Company. Cell culture reagents including RPMI 1640 medium were from Invitrogen Corp. All other reagents and chemicals used were from Sigma-Aldrich.

### Induction of hepatitis and treatment with cannabidiol

Concanavalin A was dissolved in pyrogen-free PBS at a concentration of 2.5 mg/ml and injected intravenously at a dose of 12.5 mg/kg body weight to induce hepatitis as described [54]. Mice were administered intraperitoneally with indicated doses of cannabidiol (DMSO stock diluted in PBS + a drop of Tween-80) or vehicle (DMSO similarly diluted in PBS) 5 min after ConA injection. One group received cannabidiol alone. Blood was collected after 6, 12, 24 and 48 h by retro-orbital bleeding and sera were separated and stored below −20°C until further use.

### Aspartate transaminase (AST) activity

Liver enzyme aspartate transaminase activity in sera from individual mice obtained at various time points after ConA injection was measured at 340 nm by spectrophotometric method using a commercially available AST assay kit (Pointe Sci.), as described previously [54].

### Liver histology

After 48 h of post ConA-injection, livers were harvested, carefully rinsed with PBS and fixed in 4% paraformaldehyde. Fixed liver tissue was embedded in paraffin, cut into 5 µm thick sections, deparaffinized in xylene, and serially dehydrated in decreasing concentrations of ethanol. Sections were stained with hematoxylin-eosin (H&E) and examined under light microscopy to evaluate and liver damage. Area of necrotic lesions as a percentage of total liver parenchyma was quantified using NIH ImageJ software. At least five fields per section were analyzed from each liver sample.

### Cytokine analysis

Serum obtained 12 h after ConA-injection was used for analyzing 23 different cytokines and chemokines including IL-2, TNF-α, IFN-γ, IL-1α, IL-1β, IL-3, IL-4, IL5, IL-6, IL-10, IL-12p40, IL-12p70, IL-17, GM-CSF, G-CSF, KC, MIP-1α, RANTES and eotaxin-1 by Bioplex cytokine array system (BioRad).

### Reverse Transcription-PCR (RT-PCR)

RT-PCR was performed by standard protocol. The total RNA was prepared using RNeasy kit (QIAGEN), and cDNA was prepared using random hexamer primers (Invitrogen). Gene-specific primers used for amplification were: SOCS-1 (forward 5′ GAG GTC TCC AGC CAG AAG TG 3′ and reverse 5′ CTT AAC CCG GTA CTC CGT GA 3′), SOCS-2 (forward 5′ AAG ACA TCA GCC GGG CCG ACT A 3′ and reverse 5′ GTC TTG TTG GTA AAG GTA GTC 3′), 18S (forward 5′ GCC CGA GCC GCC TGG ATA C 3′ and reverse 5′ CCG GCG GGT CAT GGG AAT AAC 3′). 18S served as the internal control. The PCR products were electrophoresed on 1.5% agarose gel in the presence of ethidium bromide and visualized in a gel imaging system (BioRad). The densities of bands were analyzed with NIH Image J software and normalized to internal control.

### Cell preparation and flow cytometric analysis

Liver infiltrating cells were isolated using Percoll density separation. Briefly, single cell suspensions from livers were prepared by using a tissue homogenizer and passing the homogenate through sterile nylon mesh (70 µM). Cell suspension was washed once with PBS and pellet suspended in 33% Percoll (Sigma-Aldrich) diluted in sterile PBS and centrifuged at 2000 rpm for 15 min at 25°C. Leukocyte cell pellet was washed twice with PBS. Contaminating RBCs were lysed using RBC-lysis solution. For FACS analysis, cells were blocked using mouse Fc-block (anti-CD16/CD32) and stained for various cell surface markers using fluorescently labeled mAb (10 µg/mL, in PBS containing 2% FBS). After washing, stained cells were analyzed in a flow cytometer (FC500, Beckman Coulter). Only live cells were counted by setting gates on forward and side scatters to exclude cell debris and dead cells. Cells stained similarly with isotype antibodies served as staining controls to set the voltage. Data obtained were analyzed in Cytomics CXP software (Beckman Coulter).

### Arginase Activity Assay

Cell lysates were obtained by suspending the cell pellet in lysis buffer (50 mM HEPES, 150 mM NaCl, 5 mM EDTA, 1 mM NaVO_4_, and 0.5% Triton X-100) containing 50 µg/ml aprotinin, 50 µg/ml leupeptin, 100 µg/ml trypsin-chymotrypsin inhibitor, and 2 mM PMSF. Lysates were centrifuged at 3000×g for 10 min at 4°C. Protein content was determined by BCA method (Sigma-Aldrich) and cell lysates (5 µg) were tested for arginase activity by measuring the production of L-ornithine. Briefly, cell lysates were added to 25 µL of Tris-HCl (50 mM; pH 7.5) containing 10 mM MnCl_2_. This mixture was heated at 55–60°C for 10 min to activate arginase. Then, a solution containing 150 µL carbonate buffer (100 mM) and 50 µL L-arginine (100 mM) was added and incubated at 37°C for 20 min. The hydrolysis reaction from L-arginine to L-ornithine was identified by a colorimetric assay after the addition of ninhydrin solution and incubation at 95 °C for 1 h.

### Wright-Giemsa staining

Cells were collected^ ^by cytospin onto glass slides and dried completely for 30 min. Slides were then stained with Wright-Giemsa stain (Fisher Sci.) according manufacturer's instructions and analyzed by light microscopy.

### Cell sorting

Mice were injected with ConA (12.5 mg/kg) + CBD (25 mg/kg). Liver infiltrating cells were isolated after 12–16 h by Percoll separation. Cells were blocked using Fc-block and stained using appropriate mAb. CD11b^+^Gr-1^+^ cells (MDSCs), CD11b^+^Ly6-G^+^Ly-6C^+^ granulocytic (Gr-MDSC) and CD11b^+^Ly6-G^−^Ly-6C^+^ monocytic (Mo-MDSC) subsets were sorted to >90% purity using FACS Aria (BD Biosci.) after labeling with appropriate fluorescently conjugated mAbs.

### T cell proliferation assay

Purified MDSCs were irradiated at approximately 2000 rads and cultured at different ratios with purified syngenic T cells (2×10^5^) stimulated with ConA (4 µg/ml) in a 96-well round bottom plates, in complete RPMI 1640 medium (Invitrogen) supplemented with 10% FBS, 10 mM HEPES, 1 mM penicillin-streptomycin, and 50 µM β-mercaptoethanol. T cell proliferation was determined after 48 h culture by pulsing with [3H]thymidine (2 µCi/well) during the final 8 h of culture. Cultures were harvested using a cell harvester and thymidine incorporation was measured in a beta counter (Perkin Elmer).

### Adoptive transfer experiments

ConA was injected into naïve C57BL/6 (WT) mice to induce hepatitis as described before. For adoptive transfer, purified CD11b^+^Gr-1^+^ MDSCs from the livers of ConA+CBD injected mice were transferred to a group of naïve mice (5×10^6^ purified MDSCs/mouse, *i.v*) 12 h before injecting ConA. Blood was collected 12 h after ConA challenge. Hepatitis was assessed by measuring liver enzyme aspartate transaminase (AST) in sera.

### Statistical Analysis

Data are expressed as mean ± S.E.M. Student's *t*-test was used for comparison and *P≤*0.05 was considered statistically significant. Experiments were repeated at least twice unless otherwise mentioned.
